# Preoperative lateral subluxation of the patella is a predictor of poor early outcome of Oxford phase-III medial unicompartmental knee arthroplasty

**DOI:** 10.3109/17453674.2011.618915

**Published:** 2011-11-24

**Authors:** Stig Munk, Anders Odgaard, Frank Madsen, Jesper Dalsgaard, Lars Peter Jorn, Otto Langhoff, Claus Fink Jepsen, Torben Bæk Hansen

**Affiliations:** ^1^Department of Orthopedics, Holstebro Regional Hospital, Hostebro; ^2^Department of Orthopedics, Aarhus University Hospital, Aarhus; ^3^Department of Orthopedics, Viborg Regional Hospital, Viborg; ^4^Department of Orthopedics, Horsens Regional Hospital, Horsens; ^5^Department of Orthopedics, Randers Regional Hospital, Randers; ^6^The Orthopaedic Research Unit, Hospital Unit West, Denmark

## Abstract

**Background and purpose:**

There is disagreement in the literature about the importance of patellofemoral joint degeneration and knee pain for the outcome of unicompartmental knee arthroplasty (UKA). We therefore investigated the importance of selected predictors including patellofemoral joint degeneration and the location of preoperative knee pain for the early outcome of UKA.

**Patients and methods:**

The study group comprised 260 consecutive patients from 5 hospitals who underwent Oxford UKA for anteromedial osteoarthritis. Data were collected at baseline and included pain location, radiologically observed degeneration of the patellofemoral joint including subluxation of the patella, intraoperative cartilage status of the patellofemoral joint, disease-specific knee status, and Oxford knee score (OKS). Outcomes were evaluated after 1 year using the OKS, global patient satisfaction, and global patient result.

**Results:**

The average OKS score at baseline was 24 (SD 7), and it was 40 (SD 8) at the 1-year follow-up. 94% of the patients claimed improvement after the operation and 90% were satisfied with the UKA. Lateral subluxation of the patella was a predictor of poor outcome, and the preoperative OKS score was also a predictor of outcome. Full-thickness cartilage loss at any location gave a similar outcome to that with a normal or near-normal joint surface, and likewise, preoperative anterior knee pain was not a predictor of outcome.

**Interpretation:**

We conclude that the good early outcome after UKA in this study is in line with the best reported results. Patellofemoral degeneration should not be considered a contraindication to Oxford UKA. Patients with lateral subluxation of the patella have an increased risk of a poor result after UKA and should preferably be offered a total knee replacement.

There is no consensus about the indications for choosing unicompartmental knee arthroplasty (UKA) instead of total knee arthroplasty. Kozinn and Scott (1989) accepted only minor degenerative changes in the patellofemoral joint, and anterior knee pain—thought to be a sign of significant patellofemoral involvement—has also been an exclusion criterion (Stern et al. 1993). Berger et al. (2004) stated that patients with clinical, radiographic, or intraoperative evidence of patellofemoral arthrosis are not appropriate candidates for unicompartmental knee arthroplasty. However, the Oxford Group recommended that the state of the patellofemoral joint should be ignored when deciding whether or not to use UKA (Goodfellow et al. 1986, 2006). A recent paper by the Oxford group (Beard et al. 2007a) demonstrated that anterior knee pain or damage to the patellofemoral joint (provided that there is not bone loss and grooving of the lateral facet) is not a contraindication for Oxford UKA, while caution should be observed in cases with lateral patellofemoral joint degeneration. In these cases, a TKR should be preferred to avoid clinical failure.

Here we describe early outcome after Oxford phase-III UKA. We also investigated the importance of selected predictors—including patellofemoral joint degeneration, subluxation of the patella, and the location of preoperative knee pain—for early outcome.

## Patients and methods

The study was a prospective cohort multicenter study, where we consecutively included all patients who were operated with a medial Oxford UKA (Biomet, Bridgend, UK) between January 2007 and December 2008 at 5 cooperating hospitals in Denmark. The patients were included according to the criteria specified by the Oxford Group (Price et al. 2001). Patients with patellofemoral instability were excluded. Patients with cognitive dysfunction and severe systemic disease with functional impairment were also excluded. It was left to the discretion of the surgeons to obtain a skyline view of the patellofemoral joint and to decide whether patients with severe patellofemoral osteoarthritis should be operated.

Data were collected at baseline before the operation and subdivided into five predictor groups: (1) hospital and patient, (2) location of pain, (3) radiographically observed degeneration including subluxation, (4) cartilage degeneration, and (5) disease-specific knee status.

### Hospital and patient characteristics

As potential predictors, we included the hospital in which the patient was operated together with gender, age, height, weight, and body mass index (BMI) where we considered high BMI to be a potential predictor of poor outcome.

### Location of pain

**Figure 1.**
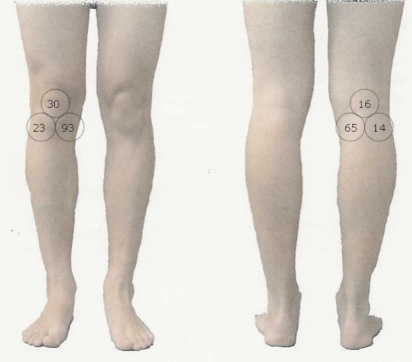
Location of pain (%) at baseline in 204 unicompartmental knee arthroplasty patients.

On a knee picture with 3 circles anteriorly and posteriorly, the patients were asked to mark their knee pain anteromedially, anterocentrally, anterolaterally, posteromedially, posterocentrally, or posterolaterally (Figure 1).

### Radiographically observed degeneration

**Figure 2.**
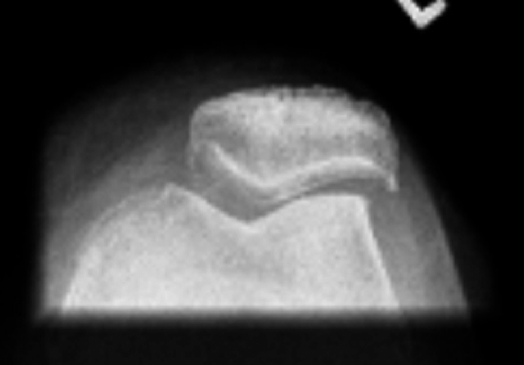
Preoperative skyline view of the patellofemoral joint with lateral subluxation of the patella.

In all 202 patients tested (202 knees), the radiographic assessment included a skyline view taken with the knee flexed between 30 and 45 degrees (Merchant 2001). Both medial and lateral aspects of the patellofemoral joint were assessed to determine the presence of degenerative changes. The radiographs were scored for severity of osteoarthritis by one observer (SM) using the Kellgren-Lawrence (1957) grading. Grade 4 (definite osteophytes with severe joint space narrowing and subchondral sclerosis) was considered to be a potential predictor of poor outcome. We also assessed whether or not the patella was laterally subluxated or laterally tilted. A lateral subluxation was defined as a positive congruence angle (Merchant et al. 1974) (Figure 2), which was considered a potential predictor of poor outcome.

### Cartilage degeneration as a predictor

The standard minimally invasive surgical procedure was used (Price et al. 2001) and no surgery was done to correct a preoperative lateral subluxation of the patella. The state of the patellofemoral joint was classified intraoperatively according to a 4-point grading system, from no cartilage loss to full-thickness cartilage loss (Weidow et al. 2002). Grade 4 (full-thickness cartilage loss) was considered a potential predictor of poor outcome. The joint was divided into 4 locations: medial patella facet, lateral patella facet, medial trochlea facet, and lateral trochlea facet. The exposure was sufficient for full inspection of the trochlear groove and the medial patellar facet, but in some cases a sufficient view of the lateral patellar facet could not be obtained.

### Disease-specific knee status as predictor

Disease-specific knee status was assessed by using the summed score from the Oxford knee score (OKS) questionnaire (Dawson et al. 1998). OKS was scored from 0 to 48, with 48 being the best possible outcome.

### Outcome

Follow-up was done 1 year after the operation. The OKS summed score was used as primary outcome measure at all time points, and both follow-up score and the change in score from baseline to follow-up were estimated. OKS questionnaires were sent to the patients 1 year after surgery, together with pre-paid reply envelopes. OKS was supplemented with the same pain drawing as used preoperatively, where the patient could make a mark if he or she still had knee pain (Figure 1). The patients were finally asked if they were satisfied with the result of the UKA operation (4 answer categories in an ordinal scale from “very satisfied” to “dissatisfied”), and how they rated their overall knee status (5 answers categorized in an ordinal scale from “the knee functions much better” to “the knee functions worse”).

To avoid having missing data, we sent a reminder with a second copy of the questionnaire if we had not received the questionnaire within 4 weeks. If we did not receive the second questionnaire within another 4 weeks, the patient was contacted by telephone.

### Statistics

Univariate and multivariate ordinary least-squares regression by stepwise model building according to Hosmer and Lemenshow (2001) was performed in order to identify preoperative predictors of outcome at 1-year follow-up. Step 1 in the multivariate analysis was a univariate analysis of all variables. Any variable that had a p-value of < 0.25 was a candidate for the multivariable model. Step 2 was a multivariate analysis including all selected candidates. Step 3 was exclusion of non-contributing variables, and fitting of new models without these non-contributing variables. The variables were excluded one at a time with the variable with the highest p-value first, until only variables with p-values of < 0.05 remained in the model. After inspection of the residuals in the preliminary final model of the multivariate linear regression, and if there was no sign of misfit, it was then considered to be the final model, which was estimated by using R^2^. All the predictor variables that remained in the final model with OKS score at follow-up were always included in the model building with OKS change score from baseline to follow-up.

## Results

### Patients

268 patients (268 knees) were eligible for the study. Of these, 8 patients were excluded at the 1-year follow-up. There were 3 revision surgeries. 1 knee was revised for early component loosening, 1 for infection, and 1 for severe cartilage injury in the lateral compartment because of retained loose cement. 2 patients were excluded because of cognitive impairment after vascular cerebral insults. 1 patient died in the follow-up period. 2 patients failed to return the OKS questionnaire.

### Hospital and patient characteristics

260 patients (mean age 66 (SD 9), 51% men) from the five hospitals were included in the study (Table 1).

**Table 1.** Baseline characteristics and included variables of 5 predictors areas in 260 unicompartmental knee arthroplasty patients

Predictor	n (%)	Mean (SD)
Hospital and patient		
Hospital		
1	38 (15)	
2	87 (33)	
3	100 (39)	
4	24 (9)	
5	11 (4)	
Sex, female	127 (49)	
Age		66. (9)
BMI		28 (5)
Height, m		1.70 (0.1)
Weight, kg		83 (160)
Radiologically observed degeneration (K-L grade 4)		
Medial	10 (5)	
Lateral	5 (3)	
Subluxation of patella	7 (3)	
Cartilage degeneration (full-thickness cartilage loss)		
Patella medial	7 (3)	
Patella lateral	4 (2)	
Troclea medial	19 (8)	
Troclea lateral	12 (5)	
Pain location		
Anteromedial	189 (93)	
Anterolateral	46 (23)	
Anterocentral	61 (30)	
Posteromedial	132 (65)	
Posterolateral	28 (14)	
Posterocentral	32 (16)	
Disease-specific knee status		
OKS at baseline		23 (7)

### Location of pain

204 patients (79%) marked the preoperative location of pain on the knee picture. These locations are presented in Figure 1 and Table 1.

### Radiographically observed degeneration

202 knees (78%) had a preoperative skyline view of the patellofemoral joint. Only 10 (5%) had severe degenerative changes in the medial part of the patellofemoral joint and 5 (3%) in the lateral part. Lateral subluxation was identified in 7 knees (3%) (congruence angle median 19° (IQR: 17–29), lateral tilt median 9° (IQR: 8–12)). 1 of the patients with lateral subluxation of the patella had severe degenerative changes in the lateral part of the patellofemoral joint (Table 1).

### Cartilage degeneration

The state of the cartilage in the patellofemoral joint was recorded intraoperatively in 253 patients (97%). Full-thickness cartilage loss on the medial site of the patella was seen in 7 patients (3%), on the lateral site of the patella in 4 patients (2%), on the medial site of the trochlea in 19 patients (8%), and on the lateral site of the trochlea in 12 patients (5%) (Table 1). 1 of the seven patients with lateral subluxation of the patella had full cartilage loss in the lateral part of the trochlea and none at the lateral patellar facet.

### Disease-specific knee status

The OKS at baseline was 24 (SD 7) (Table 1). At the 1-year follow-up, 108 patients (42%) reported persistent knee pain, but the OKS had risen to 40 (SD 8) (Table 2).

**Table 2.** Location of pain and outcome at one-year follow-up for 257 unicompartmental knee patients

Predictor	n (%)	Mean (SD)
Still having pain	108 (42)	
Pain location:		
Anteromedial	51 (48)	
Anterolateral	42 (39)	
Anterocentral	38 (36)	
Posteromedial	44 (41)	
Posterolateral	19 (18)	
Posterocentral	21 (20)	
Disease-specific knee status		
Knee status:		
0	212 (84)	
1	27 (11)	
2	7 (3)	
3	4 (2)	
4	4 (2)	
5	0	
Satisfied with result:		
0	183 (73)	
1	46 (18)	
2	21 (9)	
3	4 (2)	
4	0	
Oxford knee score at follow-up		40 (8)
Change in Oxford knee score		16 (8)

### Univariate and multivariate analysis of all predictors

*Hospital and patient characteristics. *No hospital or patient characteristics were found to be predictive of outcome at the 1-year follow-up or the change from baseline to follow-up (Tables 3 and 4).

**Table 3.** Univariate and multivariate analysis of all included predictor variables on knee status at 1-year follow-up

	Univariate analysis			Multivariate analysis **^a^**		
Predictor	Coefficient	(95% CI)	p-value	Coefficient	(95% CI)	p-value
Hospital and patient						
Hospital	–0.04	(–0.8 to 0.8)	0.9			
Sex	–0.7	(–2.6 to 1.2)	0.5			
Age	0.05	(–0.1 to 0.1)	0.3			
BMI	–0.1	(–0.3 to 0.1)	0.2			
Height	4.8	(–6.0 to 15.4)	0.4			
Weight	–0.03	(–0.1 to 0.3)	0.4			
Pain location						
Anteromedial	–3.7	(–7.7 to 0.4)	0.08			
Anterolateral	–2.9	(–5.4 to –0.3)	0.03			
Anterocentral	–1.3	(–3.5 to 1.0)	0.3			
Posteromedial	0.8	(–1.4 to 3.0)	0.5			
Posterolateral	0.4	(–2.7 to 3.4)	0.8			
Posterocentral	3.0	( 0.1 to 5.8)	0.04	3.0	(–0.3 to 5.7)	0.03
Radiolographic degeneration						
Medial	–0.1	(–6.1 to 4.2)	0.7			
Lateral	0.4	(–6.7 to 7.5)	0.9			
Subluxiation of patella	–10.3	(–16.0 to –4.5)	0.	–6.4	(–12.2 to –0.6)	0.03
Cartilage degeneration						
Patella medial	–1.0	(–6.8 to 4.8)	0.7			
Patella lateral	–2.4	(–0.0 to 5.1)	0.5			
Trochlea medial	–0.8	(–4.4 to 2.8)	0.7			
Trochlea lateral	–1.0	(–5.4 to 3.5)	07			
Disease-specific knee status						
OKS at baseline	0.4	(0.2 to 0.5)	0.00	0.4	(0.2 to 0.5)	0.00

**^a^** R^2^ = 0.17.

**Table 4.** Univariate and multivariate analysis of all included predictor variables on change in knee status at 1-year follow-up, with significant predictors shown in bold

	Univariate analysis			Multivariate analysis **^a^**		
Predictor	Coefficient	(95% CI)	p-value	Coefficient	(95% CI)	p-value
Hospital and patient						
Hospital	0.5	(–0.4 to 1.4)	0.3			
Sex	2.1	(–0.04 to 4.3)	0.05			
Age	0.05	(–0.1 to 0.1)	0.9			
BMI	0.1	(0.1 to 0.3)	0.3			
Height	–7.1	(–19.2 to 4.9)	0.2			
Weight	–0.01	(–0.1 to 0.1)	0.5			
Pain location						
Anter medial	–4.3	(–8.9 to 0.3)	0.07			
Anterolateral	–0.1	(–2.9 to 2.7)	0.9			
Anterocentral	2.4	(–0.1 to 4.9)	0.06			
Posteromedial	2.2	(–0.2 to 4.6)	0.07			
Poster lateral	3.2	(–0.2 to 6.6)	0.06			
Posterocentral	2.8	(–0.3 to 5.9)	0.08	3.0	(–0.3 to 5.7)	0.03
Radiographic degeneration						
Medial	–1.6	(–6.9 to 3.7)	0.6			
Lateral	0.6	(–6.8 to 8.0)	0.9			
Subluxation	–3.4	(–9.7 to 2.8)	0.3	–6.4	(–12.2 to –0.6)	0.03
Cartilage degeneration						
Patella medial	–2.8	(–9.1 to 3.4)	0.4			
Patella lateral	–3.8	(–12.0 to 4.4)	0.4			
Troclea medial	–2.1	(–6.0 to 1.7)	0.3			
Troclea lateral	0.01	(–4.8 to 4.8)	1.00			
Disease-specific knee status						
OKS at baseline	–0.6	(–0.8 to –0.5)	0.00	–0.6	(–0.8 to –0.5)	0.00

**^a^** R^2 ^= 0.29.

*Location of pain. *Reporting of anterolateral pain was found to be a predictor of poor outcome at follow-up in the univariate analysis (OKS –2.9 (p = 0.03)); however, it was not found to be a statistically significant predictor in the multivariate analysis. Posterocentral pain was found to be a predictor of good outcome at follow-up (OKS 3.0 (p = 0.04)) in both the univariate and the multivariate analyses (Table 3). Likewise, posterocentral pain was found to be a predictor of change in OKS from baseline to the one-year follow-up (OKS 3.0 (p = 0.03)) (Table 4).

*Radiographically observed degeneration and cartilage degeneration.* Subluxation of the patella was found to be the only predictor of poor outcome at follow-up (OKS –10.3 (p = 0.03)) in both the univariate and the multivariate analyses for OKS score at follow-up and the change in OKS score from baseline to follow-up (Tables 3 and 4).

**Figure 3.**
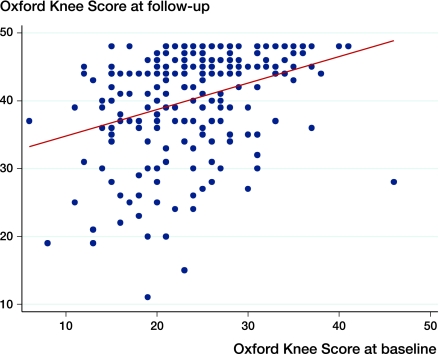
Correlation (with best-fit line) between Oxford knee score at baseline and Oxford knee score at follow-up.

**Figure 4.**
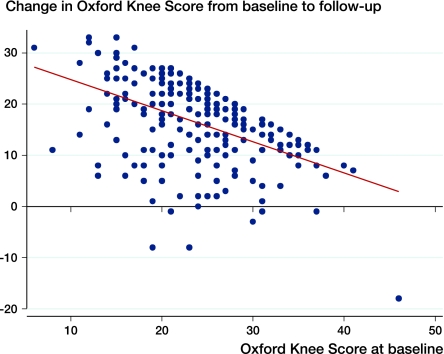
Correlation (with best-fit line) between Oxford knee score at baseline and change in Oxford knee score from baseline to follow-up

*Disease-specific knee status as a predictor. *The OKS at baseline was found to be a predictor of outcome at follow-up (OKS 0.4 (p < 0.01)) in both the univariate and multivariate analyses (Table 3 and Figure 3). The OKS at baseline was also found to be a predictor of a lower change in score from baseline to follow-up (OKS –0.6 (p < 0.01)) in both the univariate and the multivariate analyses (Figure 4).

## Discussion

In most reports on patients with medial knee osteoarthritis, anterior pain from the patellofemoral joint is not clearly distinguished from pain from the femorotibial joint (Yutaka et al. 2003), and anterior knee pain is also a poorly defined entity (Beard et al. 2007a). The Oxford Group (Beard et al. 2007a) asked their patients to specify whether their pain was medial, anterior, lateral, or generalized. 55% had preoperative anterior knee pain but only 3% had lateral pain, and they found no difference in OKS outcome between the groups with and without preoperative anterior knee pain (Beard et al. 2007a). In our study, we found that 30% of the patients had anterocentral knee pain before Oxford UKA, but the OKS outcome 1 year postoperatively was independent of the presence or absence of anterocentral preoperative pain. 23% had preoperative anterolateral knee pain, and in our study this was a predictor of poor outcome in the univariate analysis, but not in the multivariate analysis. Based on this, we agree with the Oxford group that preoperative anterior knee pain does not correlate with the result after Oxford UKA. However, they did not look at the correlation between posterior knee pain and the result after UKA. In our study, we found that 16% of the patients had preoperative posterocentral knee pain, and this was a predictor of good outcome. The reason for this is uncertain. It may be due to soft tissue-related disorders.

In a study by Seyler et al. (2009), patellar osteophytes were not found to be a predictor of outcome after UKA in a study involving 80 UKAs. Beard et al. (2007b) did not find full-thickness cartilage loss in the patellofemoral joint to be associated with a poor result after UKA, and our results are similar; in our view, there is therefore no reason to exclude these patients from UKA. The Oxford Group also assessed preoperative subluxation of the patella on the skyline radiographs, but did not report any analysis of outcome (Beard et al. 2007a). In a more recent study (Pandit et al. 2011), they claimed subluxation to be a contraindication to UKA but did not address it in the study. In our study, 7 patients had lateral subluxation of the patella on the preoperative skyline radiographs, and this was a highly significant predictor of poor outcome. Chang et al. (2007) found that lateral patellar translation of the patella and difficulty in rising from a chair was predictive of increased anterior knee pain in osteoarthritic knees after total knee replacement. They suggested that patellofemoral malaligment and abnormal tracking may be an important cause of postoperative pain. We suggest that preoperative patellofemoral malaligment or abnormal tracking will not be corrected after medial Oxford UKA, and could be a cause of inferior outcome. Stress radiography of the patellofemoral joint with and without quadriceps contraction is a simple low-cost method to evaluate patellar shift (Fukui et al 2003), and could be a screening method for lateral subluxation. Our results indicate that UKA should not be used in these patients—but a total knee arthroplasty should be used instead. A limitation of our study is that it involved multiple centers, where 12 different surgeons included patients in the study. It was left at the discretion of the surgeons to obtain a skyline view of the patellofemoral joint and 22% of the patients included had no preoperative skyline view. We had few patients with severe osteoarthritis in the patellofemoral joint on skyline radiograph, and this may have been due to selection bias in the study. In our study, the conclusions of the Oxford Group regarding the importance of severe degenerative changes in the lateral part of the patellofemoral joints for the outcome can be supplemented with the statement that preoperative anterolateral knee pain and lateral subluxation of the patella also have a negative influence on outcome of Oxford UKA.

In conclusion, the clinical outcomes in this multicenter study after UKA are in line with the best results reported. Patellofemoral degeneration should not be considered a contraindication to Oxford UKA. Patients with lateral subluxation of the patella have an increased risk of a poor result after Oxford UKA and should preferably be offered a total knee replacement instead.
